# The phenomenological method in qualitative psychology and psychiatry

**DOI:** 10.3402/qhw.v11.30682

**Published:** 2016-03-09

**Authors:** Magnus Englander

**Affiliations:** Department of Social Work, Faculty of Health and Society, Malmö University, Malmö, Sweden

**Keywords:** epochē, qualitative methods, Giorgi, Davidson

## Abstract

This article will closely examine the phenomenological method as applied to qualitative inquiry in psychology and psychiatry. In a critical comparison between Amedeo Giorgi's and Larry Davidson's qualitatively methods, conclusions were drawn with regard to how different kinds of qualitative inquiry are possible while remaining faithful to Husserlian philosophical foundations. Utilizing Lester Embree's recent articulation of how Husserl's method of the *epochē* can be disclosed as specific to a discipline, varieties of these two qualitative methods were seen in their relation to the original scientific aim instigated by the developer.

Qualitative research methods, purporting to be based upon philosophical phenomenology, have been inaccurately criticized by Shaun Gallagher (2012) who indiscriminately lumps all such approaches together stating:

Thinkers in other fields saw promise in phenomenology as a basis for qualitative research; however, it has often been the case that practitioners with only a passing knowledge of phenomenology were able to talk about “getting to” the lived experience of their clients and patients, but in some important sense they were unable to deliver. Much of their work depended on interviewing subjects about the particularities of their ongoing experiences. If questions were not framed well, however, investigators would frequently get an opinion or an explanation of why subjects were feeling a certain way rather than a description of the subjects' lived experiences. Another difficulty appeared at the point of interpretation by the investigators. Various methods for organizing the data or for developing categories that generalize the individuals' reports were brought over from psychology or the social sciences but were not necessarily phenomenological or well integrated with phenomenology. The result was that the same phenomenological data could be construed in a number of ways and could end up far removed from the lived experiences of the subjects. (p. 306)

Considering the wide variety of qualitative methods whose founders claim their investigation are phenomenological-many of which have been critiqued as inadequately philosophically grounded by phenomenological psychologists themselves (e.g., Applebaum, 2012; Giorgi, 2010)—generalizations like Gallagher's are unsurprising.

The many challenges in adopting a phenomenological philosophical approach to the sciences of psychology and psychiatry are nothing new. Close to half a century ago, Herbert Spiegelberg (1972), the eminent historian of phenomenology, noted shortcomings in adaptations of phenomenology in psychology and psychiatry. He wrote,

It is certainly true that phenomenology and existentialism have had a fatal appeal for a good many band-wagon climbers and freeloaders on the fringes of scientific psychology and psychiatry who try to profit from the prestige of the new movement by name-dropping or even without it. But this is no good reason for rejecting the legitimate claims of those who have taken serious account of the philosophical foundations of their enterprises. (p. 359)

In comparison to Gallagher's downplay of an entire research tradition, Spiegelberg's stance is better informed. In fact, the problems of certain qualitative methods referring to themselves as phenomenological have been a significant issue for those who take the phenomenological tradition to qualitative research seriously. For example, Amedeo Giorgi, recognized as the founder of the descriptive phenomenological approach to qualitative psychology, has consistently and uncompromisingly critiqued approaches to qualitative psychological methods that have not followed phenomenological criteria (for some recent critiques, see, for example, Giorgi, 2006, 2010). Hence, there are still good reasons for serious developers of qualitative research methods based on phenomenology to continue to build their methodology on solid philosophical grounds.

The purpose of this paper is to take a closer look at what constitutes a phenomenological qualitative science within psychology and psychiatry. The overall question that I raise is: from a Husserlian perspective, what philosophical phenomenological principles should underlie a qualitative research method? In a critical comparison between Amedeo Giorgi's (2009) and Larry Davidson's (2003) qualitative methods, I will claim that both represent serious and fruitful (human) scientific attempts to qualitative inquiry by remaining faithful to their Husserlian philosophical foundations. As a line of argument, I will utilize Lester Embree's (2011) recent articulation (for pedagogical reasons) of how Husserl's method of the *epochē* is specific to a discipline and suggest that qualitative methods, using phenomenology as their approach, could be articulated in differing versions depending on the original scientific aim of the developer.

## Background

As qualitative researchers in phenomenological psychology and psychiatry we do see a value in being engaged in a more broadly defined understanding of “empirical” inquiry, and this separates us from a traditional, transcendental phenomenological philosophy, which is envisioned along purely eidetic lines. At the same time, we are situated in a phenomenological theory of science, meaning that we do not follow an empirical theory of science (in the mainstream, positivistic interpretation of empiricism), which means that the term “empirical” is not referring to our theory of science (Giorgi, 2009). Even if we use the phenomenological method and end up with an eidetic generalization (Giorgi, 2009), as opposed to an empirical generalization, at a certain stage in the research process we do engage with *real* psychic events. The term empirical then becomes broadened for us to include the *irreal*. Hence, we are primarily interested in the object in its phenomenal status, because from a phenomenological perspective “the object always transcends the act in which it appears” (Giorgi, 1997, p. 237). Although I will clarify these matters further, for now it is essential to indicate that the term “empirical” does not refer to our theory of science, but instead the term is used specifically in relation to our interest in real psychic events.

In developing a qualitative method, we are also explicitly dealing with various obstacles in the logical relation between scientific aim, method, and research object. Our method, adopted from philosophy, has a direction of (logical) fit towards the study of the object; however, our aim has changed from the philosophical region to the human scientific (psychology and psychiatry). Hence, we must modify our philosophical approach in order for it to be more sensitive to issues in psychology and psychiatry (Giorgi, 2009). To accomplish such a modified approach, we will have to adhere to scientific (i.e., human scientific) criteria in order to meet the demands of the new research situation. In other words, the phenomenological philosophical method needs to be congruent with our overall aim of a qualitative human science. Of course, this will make our inquiry unique and the challenge is to clarify our framework to the rest of the scientific community. One has to remember that all science has its methodological roots in a philosophy, which is equally true for the natural sciences, and historically, it was not that long ago since we made a transition from a natural philosophy to a natural science. Our human science rests on the method developed in a phenomenological philosophy, because this particular method is better suited for disclosing the subject matter we are studying.

Another observation that I want to raise at this point, and one that becomes crucial in my comparison between Giorgi's and Davidson's approaches to qualitative research, is the difference between qualitative inquiry targeted towards recovery-oriented clinical research in psychiatry versus that of research in academic psychology. I believe that such a difference plays a certain role for Davidson's use of the transcendental reduction, which is a step that is not included in Giorgi’s method. Although Davidson (1988) specifically argues that his transcendental turn is motivated by a wish to avoid a so-called *transcendental psychologism* (to be discussed later), I will argue that his orientation towards recovery-oriented research in psychiatry also plays a crucial role in his use of the transcendental reduction. Hence, having a different scientific aim than Giorgi also motivates Davidson to utilize a different “reduction” within the phenomenological method. Nevertheless, by drawing such a comparison, I will also hope to disclose what makes a qualitative approach to inquiry definitively phenomenological (in a Husserlian sense).

Before we launch into the first step of Husserl's phenomenological method, it is essential to note that Giorgi (2009, p. 91) “favors” the version in which the *epochē* and the psychological reduction are not separated, but since Davidson (2003) clearly makes these into two separate steps, I will follow Davidson's lead in terms of the organization of this paper. In a publication by Giorgi and Giorgi (2008), there is no indication that this difference has a bearing on the first two steps, also clarified in Giorgi's choice of words in which he “favors” something, indicating a matter of preference rather than an absolute distinction with decisive methodological significance.

## The *epochē*

What then constitutes phenomenological inquiry? Despite the many misunderstandings of phenomenology both inside and outside the qualitative research tradition, the most widely accepted interpretation is that *a method* is required in order to make an inquiry of an object, that is, in terms of phenomenological inquiry, to disclose *a priori* structures of consciousness. The methodical practice referred to as the *epochē* (i.e., bracketing or suspension) is often traced to Husserl's (1964) introduction of the concept in *The Idea of Phenomenology* or/and to *Ideas I* (Husserl, 1998). Husserl (1964) wrote,

Phenomenology: this denotes a science, a system of scientific disciplines. But it also and *above all denotes a method* and an attitude of mind.… (p. 18–19, my emphasis)

Even Heidegger (1962, p. 50) stated, “The expression ‘phenomenology' signifies primarily a methodological conception.” Similarly, Merleau-Ponty (1962, p. viii) remarked that, “Phenomenology is accessible only through the phenomenological method.” Within the qualitative research tradition, Giorgi (2009, p. 98) is clear on this crucial foundation in relation to the status of the method in which he emphasizes, “No claim that an analysis is phenomenological can be made without the assumption of the attitude of the phenomenological reduction.” Hence, there seems to be no Husserlian phenomenological inquiry without the *epochē*.

But why is the *epochē* so important for phenomenological research? Davidson (2003) provides us with an elaborate account,

Like the Sirens beckoning Ulysses, we find ourselves tempted at every turn to abandon our slow-going but steady labor in the realm of experience for the lure of more accessible results through a short cut into causal explanation. As we find in most get-rich-quick schemes, however, such escapes into naturalistic causality lead inevitably to the bankruptcy of qualitative approaches. More so perhaps than other theoretical perspectives grounding qualitative methods, phenomenology is very clear on this score, and it is partly for this reason that we have chosen it. To understand experience *on* its own terms we must understand it *in* its own terms, and for this purpose we place in phenomenological “brackets” our usual notions of causality. (p. 96)

As Davidson (2003) indicates, the *epochē* provides us with a clear direction away from reification and instead discloses phenomena and intentionality within the life-world. Morley (2012) takes matters a step further as he writes,

A qualitative method alone, without an accompanying approach offered by the phenomenological *epochē*, is continuously vulnerable to defaulting back into naturalistic thinking. In other words, without a fully spelled-out phenomenological epistemology, non-phenomenological qualitative researchers too often interpret their research results within the predominant naturalist assumptions of mainstream psychology and, again, subvert their own efforts to escape reductionism. (p. 589)

Nevertheless, it is common knowledge for those familiar with the phenomenological movement that there are so many versions of phenomenology that one must speak of phenomenological *methods* as plural. However, when referring to any type of Husserlian phenomenological inquiry, “It's Always About the *Epochē*,” as Morley (2010, p. 293) has uncompromisingly stated.

What then is the *epochē* and what is its role as a method in qualitative research? Utilizing the *epochē* (or bracketing) means to adopt a phenomenological attitude. Davidson (2003) situates the *epochē* within qualitative psychology as follows,

In the case of qualitative psychology, this means turning from the reigning attitude of natural science, in which the psychic is viewed as an object of nature toward the “life-world” that is the world of everyday experience. With this shift we simply describe what we find to belong to psychological subjectivity as it appears, or is experienced, in everyday life. We find our experiences of the psychic to include not only the material-physical aspects that have been isolated and studied by natural science but also the evaluative, ethical, emotive, and aesthetic aspects that previously had been excluded from our narrow natural-scientific focus on causality. (p. 97)

Thus, it becomes clear that the *epochē* allows us to study *intentionality*, instead of *causality*. In other words, we are starting to see the direction of logical fit between method and the object of study. The external, natural laws as regulated by causality, independent of subjectivity is *not* what the phenomenological method gives us access to. In fact, this is what is bracketed.

Many times students first misinterpret bracketing as a process that leads one to assume the role of an independent, completely unbiased observer. Such an interpretation seems to reflect a naive effort to assimilate the *epochē* within a student's “epistemic comfort zone,” which is derivative of a frequently unreflectively-adopted empirical theory of science. However, as has already been observed, phenomenological human scientific inquiry is based on a phenomenological theory of science because it has a different subject matter and is situated within life-world and subjectivity, with the researcher as a participant observer (Giorgi, 1971, 2009). Utilizing the *epochē* does not mean that one forgets everything one previously knew to arrive at a kind of blank state, but rather that one brackets one's natural attitude; that is, one invites *a shift in attitude* in order to look at the subject matter (i.e., the phenomenon) in a new way. As Giorgi (1970) showed us nearly a half a century ago, recognizing that one has a different subject matter from that of the natural sciences means that one must adopt a different approach and a different scientific methodology. Phenomenological research in psychology and psychiatry does not investigate *causality* but rather *intentionality*, which is another *epistemic relation* and *that* is what demands another approach and method.

There is nothing mystical or esoteric about using the *epochē*. Embree (2011, p. 120) summarizes the method as follows: “Strictly speaking, *epochē* names a mental operation, ‘reduction' refers to a consequent change in the researcher's attitude, and ‘purification' refers to a consequent change in the thing-as-intended-to whereby something is somehow purified in some respect and thereby becomes in some respect pure.” Hence the term reduction is not to be confused with reductionism, in which one level of reality is to account for all other levels, such as, for example, attempting to explain social interactions on the basis of neurobiology. Instead, as Embree (2011, p. 121) points out “… the attitudes that *epochēs* produce reduction from are the attitudes that are automatically returned to when the specific *epochē* is relaxed.” So if I suspend or bracket the natural attitude, I have taken a step away from it (“reduction from”). The use of the *epochē* is to enlighten something: to illuminate how the phenomenon is constituted (as an essential structure). The ideal goal here is a purified seeing of essences. From the point of view of a phenomenological theory of science, there are many types of *epochēs* (*or epochai*),^1^ including the ones utilized within the natural sciences (Embree, 2011). In other words, it is possible to phenomenologize nature and to look at the natural sciences from a phenomenological theory of science. As Embree (2011) has clearly outlined for us, the physicalistic *epochē* is the *epochē* that brackets the “… acceptance of animateness, i.e., mind in the things encountered …” (p. 123), the theoretical *epochē* brackets “… the acceptance of things as ‘aesthetic' in a maximally broad signification, i.e., as objects of enjoyment and suffering” (p. 121). Thus, there is nothing mystical and esoteric about the *epochē* in phenomenological inquiry, but it should not be confused with an independent observer as envisioned by positivism.

It is important to note that I am simplifying matters here for pedagogical reasons; however, there is no doubt that demystifying the *epochē* is essential in order to train future qualitative researchers in the descriptive phenomenological method. In addition, and as we saw above, from a phenomenological theory of science, even the natural scientist could be seen as using implicit philosophical steps, such as the theoretical and the physicalistic *epochēs*, in order to enter the attitude required for the physical sciences. However, such essential attitudes in the natural sciences are rarely ever disclosed philosophically because positivism has achieved such hegemony in the sciences, so much so that science envisioned positivistically is most often mistakenly equated with science as such. Even though we are not taught about the physicalistic *epochē* and the theoretical *epochē* in our physics courses as underlying philosophical attitudes, it seems hardly unlikely that the physical sciences would be possible without such “mental operations.” Embree (2011) even suggests that a combination of disciplines could benefit from certain *epochēs* essential to their specific aim of purification. He suggests in relation to a possible combination of sociology and child psychology, that “middle-class expectations in the investigator … [could be] suspended for the sake of better grasping the outlook of the member of another class or age” (Embree, 2011, p. 125). Hence, each *epochē* leads to a type of reduction and then to a specific type of purification, and the type of *epochē* will vary depending on the scientific aim.

## The psychological *epochē* and reduction

Husserl (1977) claimed that using the phenomenological psychological reduction is the minimum methodical step needed in order for one's inquiry to be properly termed phenomenological. It is a partial reduction, meaning that one remains in the natural attitude, in which psychic events are considered real and thus empirical. Developing a qualitative method for psychology based on phenomenological philosophy means that one would, in some respect, be dealing with the empirical level, for example, interviewing real persons about their real experiences in relation to real events, and at the same time finding a way to clarify the results on an eidetic level by means of phenomenological analysis. In other words, one would seek to provide an account of the empirical (factical) dimension of participants' narratives while arriving at findings that are phenomenological. The phenomenological psychological reduction would thus be the bare minimum in order to develop a qualitative psychology based on a phenomenological theory of science. According to Giorgi (2009),

With this reduction, the objects of experience are reduced (that is, reduced to phenomena as presented), but the acts of consciousness correlated with such objects belong to a human mode of consciousness. Philosophically speaking, this reduction is not as radical as the transcendental reduction, but is more appropriate for psychological analyses of human beings since the purpose of psychology as a human science is precisely the clarification of the meanings of phenomena experienced by human persons. (p. 98)

Therefore, the phenomenological psychological reduction as a method becomes essential in order to develop a phenomenological qualitative method for psychology that remains within the limits of a human consciousness, yet is interested in phenomenological results. The psychological reduction is thus adopted throughout all steps of Giorgi's (2009) qualitative method.

The psychological reduction is also part of Davidson's (2003) approach as a second step (after the *epochē*). In his 1991 article co-authored by Cosgrove, the psychological reduction is described as follows:

Through the phenomenological-psychological reduction, we abandon our commonsense understanding of reality as consisting of objects and their causal underpinnings and adopt an appreciation of reality as consisting of the acts of experiencing itself. What may appear to be a subtle shift on the level of ontology has profound significance on the level of methodology. As a result of this reduction we take an entirely different sphere than that traditionally taken by science to constitute its subject matter. We shift our focus away from a concern with the existential status of the objects experienced to concern ourselves solely with the experiencing *of* these objects in consciousness. (Davidson & Cosgrove, 1991, p. 93)

As we can see, although the *epochē* helps us to break from the natural attitude and enter the psychological reduction, our break is only partial, that is, we do so in regard to the object, which then receives the status of (or is reduced to) a phenomenon. The intentional acts of our subjects are considered real, and hence the term empirical (as opposed to transcendental) applies to these acts.

So far we have been dealing with the presence of the psychological reduction within Giorgi's as well as in Davidson's method, however, we must also briefly account for how it has been modified to fit the context of qualitative research. Giorgi (2009, pp. 94–96) points to the modification of satisfying scientific criteria, and its relation to obtaining data from others. In addition, Giorgi (2009) renames the psychological reduction “the human scientific reduction” or sometimes even “the scientific reduction” (p. 95). Such a modification is essential because it also points to what Giorgi wants to illuminate. In his use of the term “science” Giorgi (1997, 2009) is referring to the meaning of science in its broad, inclusive sense (i.e., not restricted to natural science or human science exclusively), in which one of the criteria for science is to seek *general*, instead of *universal* knowledge, the former which Giorgi reserves for science and the latter for philosophical inquiry.

In contrast, Davidson's account of the psychological seems only philosophical at first and his view of the psychological reduction points in the direction of its incompleteness for a human scientific psychology. He writes,

A psychology of personal worlds cannot be self-sufficient or autonomous, since these worlds in turn must be grounded themselves. They remain relative to “the” world in which the subjectivity of which they are a correlate is posited, naively, to be existing. Otherwise, the relative world disclosed through the personal attitude would be taken to be “the” world in which this psychological subjectivity is understood to have its being. This would be a “transcendental psychologism,” in that what is actually relative is taken to be absolute. (Davidson, 1988, p. 11)

Although Davidson takes up the problem of a “transcendental psychologism” here, which we will return to later, for now, his approach is an attempt to ground his qualitative inquiry in the *a priori* of the transcendental and to suggest the use of the transcendental reduction as a methodological step (e.g., Davidson, 2003), which is a step that goes beyond the empirical realm embedded within the partial, psychological reduction. However, the proper transcendental turn also moves beyond the human and worldly level, meaning that qualitative inquiry risks becoming philosophical inquiry, that is, to lose its sensitivity to issues of psychology and psychiatry as a science. Is there another way to look at this? According to Embree (2011, p. 125), “… the association of two of the most famous *epochēs*, the psychological and the transcendental, with the disciplines of psychology and philosophy suggest that there might be other *epochēs* specific to other disciplines.” In other words, the many reductions proposed by Husserl throughout his writings suggest that we have several options available to us.

However, in terms of Husserlian phenomenology, we must also adhere to another methodological step, i.e., the eidetic reduction, and that takes place within the psychological reduction. To properly understand this method is also to understand the difference between studying a phenomenon (achieved through the psychological reduction) and studying a population. To be selected as a participant for a qualitative phenomenological study means that you have had an experience of the phenomenon under investigation, which is not the same as to say that you belong to a population (Giorgi, 2009). In other words, I would say that, *the psychological meaning of a phenomenon always transcends the population in which the phenomenon appears* (cf., Giorgi, 1997, p. 237). Hence (and this is essential in order to understand the meaning of “representativeness” in phenomenological qualitative research) *we are studying the phenomenon and not the population*. We do not make an empirical generalization of the empirical acts of our participants. As I have argued elsewhere, it is the phenomenon that is general and not the participants (Englander, 2012), which also indicates the difference between eidetic and empirical generalizations (Giorgi, 2009; Wertz, 2010). This is not to say that one cannot do a phenomenological study of the meaning of a population as a *type*. As emphasized earlier, it is the existential index of the object that is bracketed, not the acts, and we seek to articulate the psychological meaning of the object, not to arrive at an empirical generalization regarding the acts. Thus, the psychological reduction helps us to make use of the real acts in order to explicate the psychological meaning of the phenomenon, but we are not limited to only considering real acts in our analysis. When one has entered into the psychological reduction one will also carry out the eidetic reduction (which is accomplished by means of free imaginative variation). The eidetic reduction is the method through which we seek the essence or the invariant psychological structure of the phenomenon. Such a process of methodical imaginative varying views the empirical acts that we have in the data simply as examples among multiple possible variations of a single essential structure. In fact, a counterexample drawn from our imagination can challenge or lead us to alter the psychological structure of our phenomenon. This imaginative varying does not mean that we are going beyond the data in the sense of adding a non-given explanatory factor like a psychological theory to our analysis; instead it is a way of arriving at and critically challenging our results. It is a critical method inviting a critical attitude that ultimately leads us to general results in which the *re-presentativeness* of the meaning of the phenomenon is possible, or as it is expressed within qualitative research, the *transferability* (cf., Lincoln & Guba, 1985) of the meaning of the phenomenon.

The eidetic reduction is critical to understanding the difference between empirical and eidetic science. As already pointed out, the phenomenological method seeks purification, and the eidetic reduction leads to a clarification of the purely essential, in our case the psychological essential structure of the phenomenon. This is why we perform the psychological *epochē* in which the existential index of our object is bracketed. The aim of the eidetic *epochē* is thus different than inductive aims of the empirical sciences. Kasmier (2010, p. 37) writes, “ … the aim of the method [the eidetic *epochē*/reduction] is a purification of the type and not a discovery of the type.” Eidetic generalizations have to do with what is essential. In his Sorbonne lectures on child psychology and pedagogy, Merleau-Ponty (2010) made a similar point,

The concept of “generality” has two meanings: either the kind found when one examines a great number of cases (and thus, generality is much greater as the cases becomes more sketchy); or the generality that one obtains in returning to the core of the concrete phenomenon, in which case one is dealing with an “essential generality.” (p. 387)

We will not go any further in terms of general knowledge claims in relation to qualitative research, because it has been covered extensively elsewhere (e.g., Englander, 2012; Giorgi, 2009; Wertz, 2010).

Let us briefly return to what the psychological reduction reveals. According to Drummond (2010, p. 159), “Phenomenological psychology … is a descriptive science that takes as its subject matter the intentional directedness of consciousness to the world.” Simply put, the *epistemic relation* being investigated in qualitative phenomenological psychological research is *intentionality*. Thus intentionality has a special meaning in terms of the psychological reduction. This means that if I experience something, that experiential relation is valid, even if the empirical nature (i.e., the existential index) of the object is not. In other words, even if the object is part of a fantasy (e.g., Santa Claus), I still had an experience of something and the phenomenon that I experienced had (and still has) psychological meaning. This is why psychological meaning rests on intentionality. The empirical science in natural scientific psychology and psychiatry aims at the identification of empirical facts and causality; however, explicating the meaning-structure of the phenomenon under study, which is constituted by intentionality, is the purpose of phenomenological psychology. As has been discussed above, this aim is made possible through the psychological and the eidetic reductions.

Up to this particular point there is perhaps no *major* difference between Giorgi's and Davidson's use of Husserl's philosophical method within their qualitative, human scientific paradigms, although their modifications and concern in regard to the psychological reduction differ. One also has to acknowledge that Giorgi's method is much more rigorous in how he specifically systematizes and analyzes all the interview data. One can only assume that Davidson goes through similar scientific procedures, although it is not spelled out. In summary then, Giorgi's modification is a psychological reduction that is sensitive to psychology as a science and results that are general, whereas Davidson interprets the psychological reduction within a personalistic attitude and finds it incomplete. Nevertheless, in these methodologists' references to each other's work there is no doubt that they are mostly working in congruence with each other's efforts.

## The transcendental *epochē* and reduction

Husserlian methodology is always difficult to sort out, and as Luft (2011, p. 52) noted, “Anybody attempting to give an account of Husserl's method of the phenomenological reduction finds oneself in an ungratifying position.” This seems especially true when one attempts to account for the transcendental reduction. Nevertheless, the transcendental reduction along with the eidetic reduction is often what we think of when we refer to Husserlian phenomenological philosophy, and the aim to purify the essence of consciousness-the intersubjective *a priori*. Embree (2011, p. 125) has captured the importance of the transcendental *epochē* for philosophy as follows, “The worldliness of minds can be suspended through transcendental *epochē* and then minds are gained in a non-worldly or transcendental status.” For Giorgi, to follow Husserl's transcendental *epochē* would mean doing philosophy rather than psychology (Giorgi 2009, p. 94). The difference between philosophy and psychology is portrayed as follows by Giorgi (2009),

When seeking essences, philosophers always seek the most universal essence, that is, those characteristics without which the object would not be what it is. Universalizing in such a way transcends psychological interest. It represents a philosophical understanding of a psychological phenomenon but without the pertinent psychological dynamics or precise uncovering of the psychological nature of the phenomenon. For example, one could say that learning always involves doing or understanding something new. That statement is essentially true, but completely nonrevelatory about the psychology of learning. To understand the living of a learning experience one has to relate correct performances to errors as well as the emotional reactions to the errors. He must understand the motivation to initiate the learning and whether that motivation was self- posited or not, the consequences of failing to learn (if that happens), and the satisfactions involved in succeeding to learn and their consequences, if that takes place. For such reasons, the universal essence is not the best way of presenting psychological results. Rather, the claim that the researcher make for the structures obtained is that they are general in the sense that the findings transcend the situation in which they were obtained. (p. 101)

Giorgi's stance makes a lot of sense, because there are obvious reasons for the science of psychology for not taking the transcendental turn, particularly because Giorgi is not seeking to illuminate the non-worldly and non-human structures of consciousness. Giorgi's (1970) aim has always been science (in its broad sense) and not philosophy.

Davidson (2003), who conducts psychological research in the context of psychiatry, especially in recovery-oriented psychiatric research, has a theoretical reason to go beyond the psychological reduction. Drawing from an extensive study of Husserl's entire corpus in terms of the argument against *psychologism*, Davidson (1988) concludes that the transcendental turn is the way to ground a human scientific psychology. Davidson (1988, p. 13) writes, “Only this grounding can allow us to overcome the transcendental psychologism of an autonomous and self-grounding phenomenological psychology.” But what is then meant by a *transcendental psychologism*? Davidson and Cosgrove (2003) write,

Remaining tied implicitly to its naturalistic heritage, a pre-transcendental psychology assumes that the objective world provides the ground for individual psychological subjects and does not recognize that transcendental subjectivity is that which constitutes both psychological subjects *and* their world. It is for this reason, among others, that psychology cannot remain transcendentally naive. (pp. 144–145)

The gist of the Davidson and Cosgrove's (2003) argument is that a pre-transcendental phenomenological psychology is naive because it is not taking stock of the transcendental constitution. Stopping at the pre-transcendental we risk seeing individual experience in terms of a background of a naive understanding of the world, similar to therapists who typically try to change “the abnormal” into “the normal” (Davidson & Cosgrove, 2003, pp. 143–144).

Let us consider an example from Davidson's (2003) study on *Living Outside Mental Illness*. If the researcher finds in the lived experience of schizophrenia a constituent, for example, “the experience of wanting to withdraw from people” in the data, it is because in the naive sense of the world, wanting to withdraw from people is abnormal. Hence in a response to the clinical psychologist or psychiatrist, there is an attempt at wanting to change the person so that she fits the ideal “normal” world again (Davidson & Cosgrove, 2003, pp. 143–144). Following Davidson's reasoning, there is a danger of stopping at the partial, psychological reduction (e.g., describing the person as withdrawing and thus experiencing a lack of motivation), because the inquiry will have its ground in a naive understanding of the world. Instead, if we take the transcendental turn we could (after having utilized our eidetic variations) see constitutions of meaning, like those in *any* serious human illness or crisis, and thus liberate the psychological subject from the stigma of *pathology* and thus situate our findings at the level of transcendental intersubjectivity. When we then return to the psychological, that is, step four in Davidson and Cosgrove's (2003) method, we could see that it is a psychological subject in a human crisis (much of what is an “invisible struggle”) that is withdrawing in order to preserve what is experientially left of the self (see, for example, Davidson, 2003, p. 153). Now, does this mean that Giorgi's method is guilty of *transcendental psychologism*? In a reply to Davidson and Cosgrove's (2003) objection to halting at the psychological reduction, Giorgi and Giorgi (2008) offer the following:

They certainly make this claim for “existential-hermeneutic” types of phenomenological psychology, but it is not clear whether they would include our type of descriptive pre-transcendental analyses. In any case, it would be erroneous to include our method under their objection because it is clear from many descriptions of phenomena that references to meanings beyond the psychological subject providing the description are clearly ascertainable. (p. 15)

Giorgi and Giorgi (2008) then provides an example, clearly showing that one can stay within the psychological reduction and still see how meanings are constituted intersubjectively and that goes beyond the subject's own experience (e.g., within cultural and social levels). In other words, there seem to be agreement in what is possible through the phenomenological approach, but the way the reductions are portrayed, interpreted, and what is actually possible to accomplish scientifically seem to differ.

In the same response to Davidson and Cosgrove (2003), Giorgi and Giorgi (2008) provide the following critical account of the use and justification of the transcendental *epochē* as a means to aim for the non-worldly and non-human. Giorgi and Giorgi (2008) write,

… while the theoretical articulation of the transcendental perspective by the authors is clear, one wonders if in the analysis the authors have gone beyond the human intersubjective world. The transcendental perspective, if intersubjective, is nevertheless beyond the human intersubjective level. The authors trace the constitutional achievements back to familial, social, and cultural factors, and thus beyond the personal, but it is not clear that the humanness has been transcended. Still, the authors ought to be commended for attempting to implement the most radical Husserlian position. (Giorgi & Giorgi, 2008, pp. 14–15)

Therefore, Giorgi and Giorgi (2008) point to the possibility that Davidson and Cosgrove (2003) are not attempting a full transcendental *epochē* and reduction. In addition, they also point to another difference in terms of interpreting the difference between the psychological and the transcendental. Giorgi and Giorgi (2008) write,

The authors stress the constituting activity of the transcendental “I,” but in our view, the psychological subject also can constitute meanings and these should not be overlooked. True, the psychological subject is the self- objectification of transcendental subjectivity, but it is a constituted subjectivity that is still capable of constituting. It is both constituted and constituting. (p. 15)

Such a clarification of the psychological level could indicate that the difference between these two methods, in terms of the actual results, might not be as radical as they seem at the theoretical level of articulation. There is a possibility that it is human intersubjectivity, which is what Davidson is aiming at but because of his interpretation of the limits of the pre-transcendental he cannot see how he can find and justify human intersubjectivity without turning to the transcendental reduction. Giorgi, on the other hand, seems to justify human intersubjectivity within his interpretation of the psychological reduction. Even though the two tracks might seem to intersect at this point, one could also suggest that they are parallel, simply because the two methodologists have different aims. Let us take a closer look at this possibility.

Davidson's attempt to make the transcendental turn seems to be motivated to guarantee the suspension of pathology in order to broaden our view of the human condition. Such a scientific aim is consistent with the science of recovery-oriented psychiatry, a science that is still battling the moralism that surrounds a naive view of mental illness (not to mention the stigma it leaves behind). More specifically, Davidson's attempt is situated in *transcendental intersubjectivity*, but also in *transcendental personalism* as present in Husserl's *Ideas II* (e.g., Kohak, 1978). Drawing from Kohak's (1978) analyses of Husserl's work, and introducing the notion of the *Person*, as the transcendental “I,” it becomes evident that Davidson's approach also refers to a *transcendental personalism* (p. 190). Davidson and Solomon (2010) write,

We must remember that this Person is not the psychological ego or human being *per se*, but is a Person equally present in its self- interpretations as psychological, historical, biological, etc. It is a Person equally as social as it is individual, equally as temporal and historical as it is spatial and material-physical. But it is also a Person whose life is not exhausted by the sum total of these varying perspectives on it. It is the living Person who takes on this number of varying modes of objective appearance, who provides the conditions for the very possibility of appearing in these ways, but who also is preserved as the source of constituting, of life, itself. It is, perhaps most accurately stated, the *life* of the Person who lives in certain objectively definable ways. (p. 106, emphasis in original)

Thus, even though some might object to Davidson not making the full transcendental turn, by his radical attempt, he is making a reduction that is different from that of Giorgi, because it is a reduction that seeks to *guarantee* to bracket pathology and thus be congruent with a specific scientific aim belonging to the science of a recovery-oriented psychiatry.

Following the recovery movement's goal to change the world, as opposed to the clinician changing the person to a set norm (see, for example, Davidson & Cosgrove, 2003, pp. 143–144), Davidson's work belongs in the overall fight for the civil rights movement of the mentally ill. Davidson's published work, situated within the recovery movement in psychiatry, clearly points in favor of such an interpretation (see, for example, Davidson, 2003; Davidson, Rakfeldt, & Strauss, 2010; Davidson, Tondora, Staehli Lawless, O'Connell, & Rowe, 2009). It comes as no surprise then that Davidson's fourth step indicates, after his transcendental turn, “a return to positivity” (Davidson & Cosgrove, 2003). As we indicated with the example from Davidson's (2003) study (on the recovery of schizophrenia) above, the fourth step includes a liberation of the psychological subject. Davidson and Solomon (2010) write,

Husserl sees the resolution of the cultural crisis of his day to reside in the active pursuit of this kind of transformative science; a science that encourages Persons to take active responsibility for themselves and the world in which they live. Viewing the world simply as an accumulation of meaningless and dead facts, already determined in advance, leaves one powerless to change it. Viewing it as meaningful and contingent upon one's intentional constitution motivates one to be responsible for it and to take an active role in trying to change it. Grounding psychology in a transcendental framework thus not only brings value and meaning back into science (through the re-appropriation of the life-world), but, just as importantly, brings science back into the on-going life of the culture. (p. 119)

Attempting to change the world instead of the patient is clearly part of the recovery movement in psychiatry and hints at a strong value statement driving scientific praxis. Such an agenda always risks mixing up science with politics, meaning that one has to tread carefully. Nevertheless, bracketing pathology in order to disclose the transcendental person also shows a great promise for the science of psychiatry by initiating a break from the traditional, cultural norms (as a naive view of the world), especially as it is embedded in our persistent stigmatization of mental illness. Recovery-oriented research, as conducted by Davidson (2003), has clearly shown that *pathology* (as in psycho*pathology*) is loaded with traditionalism, and what better way to illuminate the essence of the human condition within the lived experience of mental illness and recovery then by utilizing a type of transcendental reduction, before returning back to positivity.

Hence, the difference between Giorgi's and Davidson's strategies to qualitative research is not just in their theoretical articulations of Husserlian reductions, but how these reductions fit their aim of their particular human science. Whereas Giorgi is doing science for the sake of psychology as a rigorous human science, Davidson is adding another aim, that is, to enlighten and to broaden the human condition to get a scientific sense of mental illness, and by so doing, I would say that he is establishing a qualitative approach to a phenomenological psychiatry. Now, this is not to say that Giorgi's method would not be able to be used within the context of researching psychiatric phenomena, but to indicate that there is a difference in terms of aim compared to Davidson's approach. Hence adopting Husserlian philosophical methods to qualitative research strategies means that one has to make the necessary modifications to a reduction in order to fit one's scientific aim.

There is little doubt that the methods are “complementary” or “parallel” and represents “strong versions” of Husserlian phenomenology as applied to qualitative research strategies and human science methodology (Giorgi & Giorgi, 2008, pp. 18–19). As Giorgi and Giorgi (2008) point out,

The pre-transcendental method stays closer to the psychological phenomena but lacks complete grounding. The transcendental method is well grounded, but it tends to take in a lot more than just the psychological in order to uncover the psychological. Also, the borderline between the transcendental and the psychological has to be better understood. (p. 19)

Hence, we can then conclude that even though both of these methods are well grounded in Husserlian phenomenology, we still need to further our understanding of the boundaries between transcendental intersubjectivity and human intersubjectivity and its meaning for qualitative research in psychology and psychiatry.

Although the difference between these two qualitative methods can be seen in relation to their use of different reductions, if one probes deeper there is also an implicit difference between these two methods that can be traced to different (although complementary and parallel) scientific aims in terms of a qualitative psychology and a qualitative psychiatry. In other words, even if these two methods are different, they fall within the common scientific project of a human science. If anything, they are variations that are creatively productive and mutually supportive.

## Conclusion

Spiegelberg (1972, p. xxxvi), once stated in his now classic work *Phenomenology in Psychology and Psychiatry*, that a “luxuriant field like ours had better not be cluttered by too many varieties and subdivisions which may even interfere with our growth.” Nevertheless, and even if we should take Spiegelberg's warning to heart, we need to respect methods that have a strong foothold in their philosophical underpinnings and that can work side by side in a complementary and parallel fashion. Digging deeper than the methodological differences manifested on the surface, one can clearly find other types of reductions within Husserl's writings that could work for specific scientific aims. In other words, the warning here should perhaps not be about the variety and subdivisions, but instead about whether there is legitimate grounding in phenomenological philosophy as a methodology. Hence, staying close to the philosophical foundations is the only way to establish a qualitative, human scientific method. At this point, there should be no doubt that both Giorgi and Davidson are attempting to do just that.

## Footnotes

1 The correct plural of *epochē* is *epochai*, however, I will continue to follow Lester Embree's (2011) version here, i.e., *epochēs*, in order to be consistent with his paper *Seven Epochēs*.
